# Prognostic Factors in Stage IV Colorectal Cancer Patients With Resection of Liver and/or Pulmonary Metastases: A Population-Based Cohort Study

**DOI:** 10.3389/fonc.2022.850937

**Published:** 2022-03-15

**Authors:** Panxin Peng, Yusong Luan, Peng Sun, Liming Wang, Xufeng Zeng, Yangyang Wang, Xuhao Cai, Peide Ren, Yonggang Yu, Qi Liu, Haoyue Ma, Huijing Chang, Bolun Song, Xiaohua Fan, Yinggang Chen

**Affiliations:** ^1^ Department of Gastrointestinal Surgery, National Cancer Center/National Clinical Research Center for Cancer/Cancer Hospital and Shenzhen Hospital, Chinese Academy of Medical Sciences and Peking Union Medical College, Shenzhen, China; ^2^ Department of Clinical Medicine, Changsha Medical University, Changsha, China

**Keywords:** colorectal cancer, liver metastases, pulmonary metastases, surgical resection, prognostic factors

## Abstract

**Importance:**

Currently, surgical resection of distant metastatic lesions has become the preferred treatment for select colorectal cancer (CRC) patients with liver metastasis (LM) and/or pulmonary metastasis (PM). Metastasectomy is the most common curative method. However, evidence of the factors affecting the prognosis of CRC patients after resection of LM and/or PM is still insufficient.

**Objective:**

To explore the prognostic factors of CRC patients with LM and/or PM who have undergone resection of metastatic tumors and to provide reliable selection factors for surgical treatment in patients affected by LM and/or PM from CRC.

**Methods:**

The SEER database was used to identify eligible CRC LM and/or PM patients who underwent resection of the primary tumor and distant metastases from January 1, 2010, to December 31, 2018. The Kaplan–Meier method was used to calculate survival, and comparisons were performed using the log-rank test for univariate analysis. A Cox proportional hazards regression model was used to identify prognostic factors for the multivariate analysis. The outcomes included overall survival (OS) and cancer-specific survival (CSS).

**Results:**

A total of 3,003 eligible colorectal cancer patients with LM and/or PM were included in this study. The 3-year and 5-year OS rates were 53% and 33.6%, respectively, and the 3-year and 5-year CSS rates were 54.2% and 35.3%, respectively. In the adjusted multivariate analysis, age < 65 years (OS: p=0.002, CSS: p=0.002) was associated with better long-term outcomes, and primary tumors located on the left side of the colon (OS: p=0.004, CSS: p=0.006) or rectum (OS: p=0.004, CSS: p=0.006), T3 stage (OS: p<0.001, CSS: p<0.001), number of regional lymph nodes examined ≥ 12 (OS: p<0.001, CSS: p=0.001), and CRC LM (OS: p<0.001, CSS: p<0.001) were positive prognostic factors for survival after resection of metastatic tumors.

**Conclusion:**

Age < 65 years is associated with better long-term outcomes in colorectal cancer patients with LM and/or PM, analogously to the left sided primary tumor, T3 stage, number of regional lymph nodes examined ≥ 12 and liver metastases.

## Introduction

Approximately 149,500 cases of colorectal cancer (CRC) are diagnosed each year in the United States (1). Over half will develop distant metastases, and the liver and lung are the dominant metastatic sites. In the past decade, with the advent of new drugs and the advancement of medical technologies, survival for metastatic CRC has significantly improved. However, surgical resection is still the most likely curative method for patients with potentially resectable liver metastasis (LM). In previous surgical case series, the five-year survival rates of CRC LM patients after resection ranged from 24%-58%, with an average of 40%, and surgical mortality rates were generally<5% (2–4). There is increasing evidence that pulmonary metastasectomy can also improve the outcomes of CRC pulmonary metastasis (PM) patients (5–7). A study that included 785 CRC PM patients undergoing resection of PM with curative intent found that the 5-year overall survival rate was 68% (8), and the 5-year survival rate for patients who were treated with chemotherapy alone was at most 20% (9). Currently, surgical resection has become the preferred treatment for many appropriately selected CRC LM and/or PM patients.

Nevertheless, many factors may affect the prognosis of CRC LM and/or PM patients after surgical resection, such as age, sex, race, comorbidities, primary tumor location, primary tumor size, TNM staging, extent of distant metastasis, preoperative or postoperative chemotherapy, and radiotherapy. Identifying the clinical factors that influence patient prognosis is important for formulating reasonable treatment plans, assessing prognosis and improving the survival rate. This population-based cohort study is the first to use the SEER (The Surveillance, Epidemiology, and End Results) database to explore the prognostic factors of CRC patients with LM and/or PM who underwent resection of distant metastases with the aim of providing reliable selection factors for surgical treatment in patients affected by LM and/or PM from CRC.

## Materials And Methods

### Patients and Data Sources

This is a population-based cohort study investigating the prognostic factors of CRC patients with LM and/or PM who underwent resection of LM and/or PM. All data were obtained from the SEER database [Incidence-SEER Research Data, 18 Registries, Nov 2020 Sub (2000-2018)]. The following inclusion criteria were used: 1) stage IV CRC patients with LM and/or PM who had primary tumors and metastatic tumors resected from January 1, 2010, to December 31, 2018; 2) malignant tumor confirmed by postoperative pathology to be histological type code 8140/3 (adenocarcinoma); 3) distant metastasis proven by postoperative pathology; and 4) complete postoperative follow-up data. Exclusion criteria were as follows: 1) age <18 years; 2) a second primary cancer; and 3) distant metastases at sites other than the liver and lung, such as peritoneal, bone and brain metastases. Because the SEER database is a public database, institutional ethical approval and informed consent were not required.

### Data Collection

Demographic data included age, sex, race, primary tumor location, T stage, N stage, primary tumor size, number of primary tumor regional lymph nodes examined, distant metastatic sites, survival status, cause of death and follow-up time. Patients were categorized according to age (<65 years and ≥ 65 years), primary tumor size (≤40 mm and >40 mm), primary tumor location (right side of the colon, left side of the colon, and rectum), the number of regional lymph nodes examined (<12, 12-20, and > 20), and the presence of LM, PM, or both. All the above variables were considered important factors that may affect the outcome of CRC patients with LM and/or PM after surgical resection. After statistical analysis, the relationship between these variables and patient prognosis was explored.

### Outcomes and Statistical Analysis

The outcome endpoints included overall survival (OS) and cancer-specific survival (CSS). OS was defined as the time from resection of CRC LM and/or PM until death from any cause, and CSS was defined as the interval from resection of CRC LM and/or PM until death from cancer cause. Complete follow-up information about vital status in the SEER database was available up to December 31, 2018. Final study analyses were performed on December 01, 2021.

The survival analysis was performed using the Kaplan–Meier method, and comparisons were made using the log-rank test for univariate analysis. Variables with p < 0.1 were included in the multivariable analysis. A Cox proportional hazards regression model for multivariate analysis was used to identify prognostic factors, and a P value < 0.05 was considered a significant difference. All analyses were performed using R statistical software version 3.4.1.

## Results

**Figure 1:**
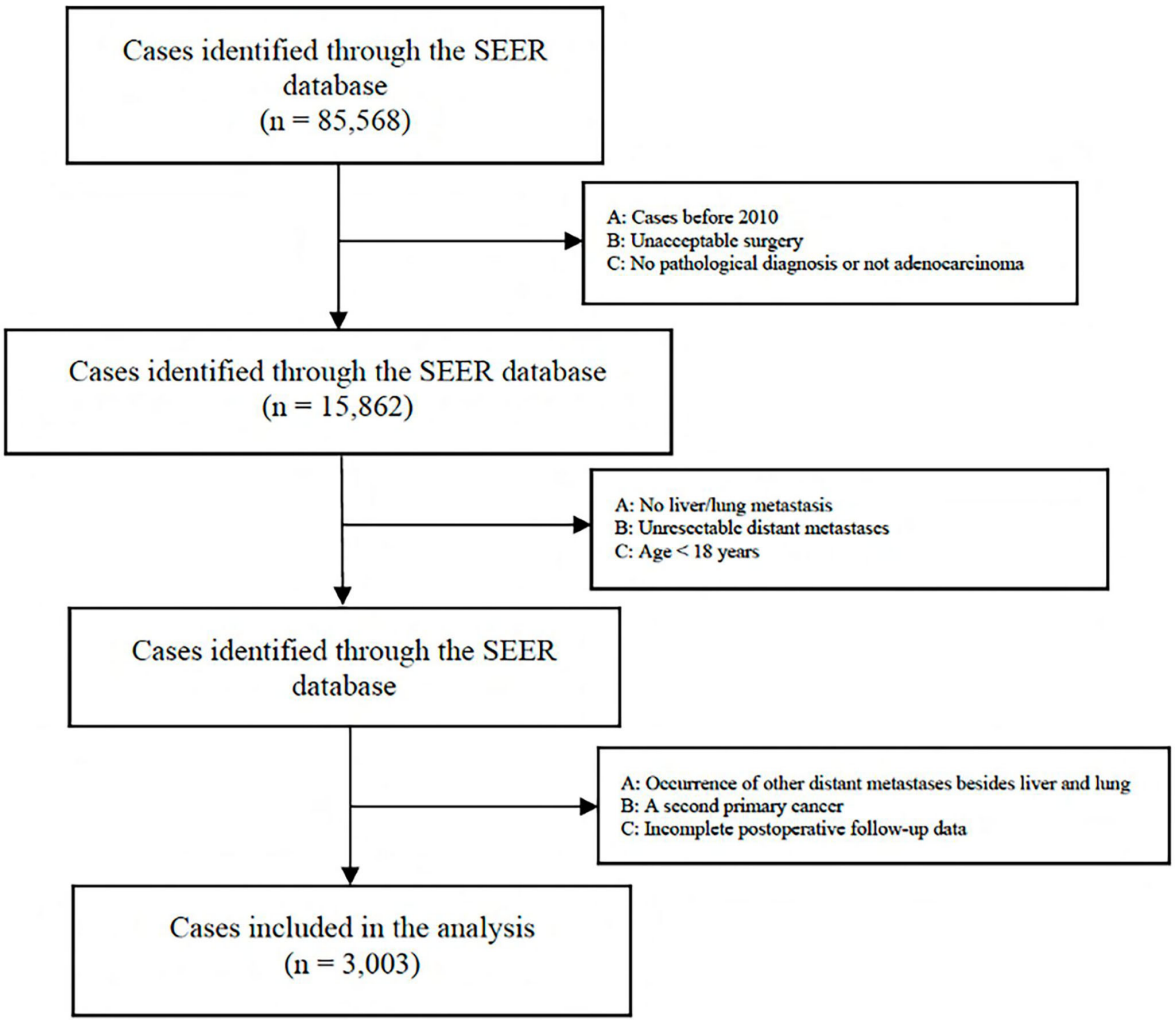
Flowchart of the patient selection.

A total of 85,568 cases were retrieved initially through the SEER database. According to our inclusion and exclusion criteria, the data of 3,003 eligible cases with IV stage CRC LM and/or PM were ultimately analyzed (**Figure 1**) . All patients underwent surgical resection of the primary tumor and metastatic tumor from January 1, 2010, to December 31, 2018. The characteristics of the patients involved in the study are shown in **Table 1**. The median follow-up time after liver and/or pulmonary metastasectomy was 21 months. Patient age ranged from 18 to 85 years old. Sixty-five percent (1950) were younger than 65 years, and thirty-five percent (1053) were 65 years or older, with 44.5% of patients being female (1336). Patients with only liver metastases accounted for 88.4%, only lung metastases accounted for 3.9%, and both liver and lung metastases accounted for 7.7%. Patients with synchronous or metachronous metastases were included in the analysis.

**Table 1:** Characteristics of the patients.

Variable	No. (%)
Age (yr.)	
<65	1950 (64.94)
≥65	1053 (35.06)
Sex	
Female	1336 (44.50)
Male	1667 (55.50)
Race	
Black	393 (13.08)
White	2318 (77.20)
Others	292 (9.72)
Primary tumor location	
Right side of colon	789 (26.28)
Left side of colon	1505 (50.11)
Rectum	486 (16.20)
N/A	223 (7.42)
T stage	
T1-2	124 (4.12)
T3	1467 (48.86)
T4	652 (21.71)
N/A	760 (25.31)
N stage	
N0	466 (15.53)
N1	995 (33.12)
N2	826 (27.52)
N/A	716 (23.83)
Primary tumor size (mm)	
≤40	321 (10.69)
>40	581 (19.35)
N/A	2101 (69.97)
Regional nodes examined	
<12	454 (15.11)
≥12, <20	1364 (45.41)
≥20	1185 (39.48)
Metastasis	
Lung	118 (3.94)
Liver	2654 (88.37)
Both	231 (7.69)

N/A Not Applicable, missing values or not specially stated.

**Figure 2:**
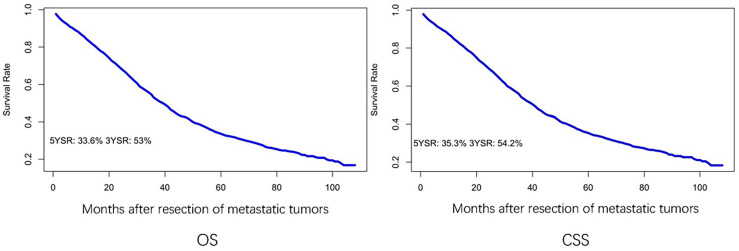
Kaplan–Meier curve of OS and CSS after resection of LM and/or PM in CRC. YSR, year survival rate.

Overall survival and cancer-specific survival curves are shown in **Figure 2**. The 3-year and 5-year OS rates were 53% and 33.6%, respectively, and the 3-year and 5-year CSS rates were 54.2% and 35.3%, respectively. In univariate analysis, age, sex, primary tumor location, T stage, number of regional lymph nodes examined, and distant metastatic sites were significant prognostic factors (**Table 2**). All these variables were included in the multivariate analysis.

**Table 2:** Univariate analysis of the prognostic factors of OS and CSS after resection of LM and/or PM in CRC.

Variable	OS			CSS		
	HR	95%CI	P Value	HR	95%CI	P Value
Age (yr.)						
<65	1	Reference	0.002	1	Reference	0.001
≥65	1.171	1.058-1.295		1.18	1.062-1.311	
Sex						
Female	1	Reference	0.072	1	Reference	0.046
Male	0.917	0.832-1.009		0.905	0.819-0.999	
Race						
Black	1	Reference	0.402	1	Reference	0.766
White	0.911	0.789-1.053		0.954	0.819-1.11	
Others	0.902	0.737-1.103		0.931	0.755-1.147	
Primary tumor location						
Right side of colon	1	Reference	0.002	1	Reference	0.006
Left side of colon	0.847	0.753-0.953		0.863	0.763-0.975	
Rectum	0.79	0.68-0.918		0.788	0.674-0.922	
T stage						
T1-2	1	Reference	<0.001	1	Reference	<0.001
T3	0.305	0.216-0.43		0.298	0.207-0.428	
T4	0.619	0.433-0.886		0.619	0.424-0.903	
N stage						
N0	1	Reference	0.1	1	Reference	0.125
N1	1	0.873-1.146		1.002	0.87-1.154	
N2	1.121	0.972-1.293		1.122	0.967-1.3	
Primary tumor size						
≤40	1	Reference	0.159	1	Reference	0.146
>40	1.159	0.935-1.437		1.173	0.939-1.465	
Regional nodes examined						
<12	1	Reference	<0.001	1	Reference	<0.001
≥12, <20	0.868	0.764-0.986		0.875	0.766-0.999	
≥20	0.811	0.711-0.925		0.805	0.702-0.924	
Metastasis						
Lung	1	Reference	<0.001	1	Reference	<0.001
Liver	0.539	0.442-0.658		0.532	0.433-0.653	
Both	3.33	1.983-5.591		3.433	2.002-5.887	

In the adjusted multivariate analysis, age, primary tumor location, number of regional lymph nodes examined, and distant metastatic sites were important prognostic factors for survival (**Table 3**). An age< 65 years was associated with better long-term outcomes (OS: HR=1.173, 95% CI 1.062 to 1.295, p=0.002 and CSS: HR=1.182, 95% CI 1.067 to 1.31, p=0.002). Patients with left-sided colon (OS: HR=0.845, 95% CI 0.754 to 0.946, p=0.004 and CSS: HR=0.861, 95% CI 0.765 to 0.969, p=0.006) and rectal tumors (OS: HR=0.787, 95% CI 0.677 to 0.916, p=0.004, and CSS: HR=0.786, 95% CI 0.67 to 0.921, p=0.006) who underwent surgical resection of metastatic tumors had a better prognosis than those with right-sided colon tumors. Patients with stage T3 disease had better long-term survival outcomes (OS: HR=0.291, 95% CI 0.234 to 0.363, p<0.001 and CSS: HR=0.284, 95% CI 0.225 to 0.358, p<0.001). The number of regional lymph nodes examined appeared to be positively correlated with long-term outcomes (OS: HR=0.829, 95% CI 0.725 to 0.949, p<0.001). Compared with PM only or both PM and LM, patients with LM only had a better long-term prognosis (OS: HR=0.56, 95% CI 0.447 to 0.657, p<0.001 and CSS: HR=0.284, 95% CI 0.225 to 0.358, p<0.001). In addition, male sex was a favorable factor for a prolonged CSS (CSS: HR=0.904, 95% CI 0.818 to 0.998, p=0.047).

**Table 3:** Multivariate analysis of the prognostic factors of OS and CSS after resection of LM and/or PM in CRC.

Variable	OS			CSS		
	Multivariate Analysis			Multivariate Analysis		
	5-y, %	HR (95%CI)	P Value	5-y, %	HR (95%CI)	P Value
Age (yr.)						
<65	36	1[Reference]	0.002	37	1[Reference]	0.002
≥65	30	1.173 (1.062-1.295)		31	1.182 (1.067-1.31)	
Sex						
Female	32	1[Reference]	0.073	33	1[Reference]	0.047
Male	35	0.916 (0.832-1.008)		37	0.904 (0.818-0.998)	
Primary tumor location						
Right side of colon	29	1[Reference]	0.004	31	1[Reference]	0.006
Left side of colon	30	0.845 (0.754-0.946)		30	0.861 (0.765-0.969)	
Rectum	35	0.787 (0.677-0.916)		37	0.786 (0.67-0.921)	
T stage						
T1-2	4	1[Reference]	<0.001	5	1[Reference]	<0.001
T3	40	0.291 (0.234-0.363)		42	0.284 (0.225-0.358)	
T4	15	0.607 (0.483-0.763)		16	0.607 (0.477-0.771)	
Regional nodes examined						
<12	27	1[Reference]	<0.001	28	1[Reference]	0.001
≥12, <20	33	0.829 (0.725-0.949)		35	0.843 (0.732-0.971)	
≥20	37	0.752 (0.654-0.866)		39	0.748 (0.646-0.867)	
Metastasis						
Lung	15	1[Reference]	<0.001	38	1[Reference]	<0.001
Liver	37	0.56 (0.477-0.657)		0	0.549 (0.466-0.647)	
Both	0	4.059 (3.114-5.29)		16	4.22 (3.217-5.537)	

## Discussion

This study is the first to use the SEER database to explore the prognostic factors of CRC patients with LM and/or PM who underwent resection of metastatic tumors. The current study showed that age, primary tumor location, T stage, number of regional lymph nodes examined, and distant metastatic sites are the most important prognostic factors. Compared with elderly patients (≥65 years), younger patients (<65 years) have better long-term outcomes. However, there is still a substantial proportion of elderly patients who have favorable long-term survival. Patients with primary tumors located in the left colon or rectum can obtain better CSS and OS after resection of metastatic tumors. It should be noted that preoperative T stage was found to be correlated with prognosis in the study; however, when the primary tumor was stage T3, patients achieved better long-term outcomes. In addition, the number of regional lymph nodes examined appears to be positively correlated with prognosis. When the number of regional lymph nodes examined is not less than 12, this may indicate a favorable prognosis. Finally, the prognosis of patients varies significantly depending on distant metastatic site. Compared with CRC patients with PM only or both PM and LM, patients with only LM have better long-term outcomes.

Previous studies have shown that age is an important factor affecting the prognosis of patients after resection of LM and PM, and advanced age (≥65 years) will increase the risk of death associated with surgery (4, 10–12). Despite this, there are still a significant number of elderly patients who can benefit from surgical resection and achieve good long-term survival. Advanced age is not an absolute contraindication for patients with CRC LM (10, 12, 13). Based on the results of our study, for elderly patients with CRC LM and/or PM, a detailed evaluation should be carried out before resection of metastatic tumors to minimize the risks of surgery and to provide elderly patients with the most appropriate treatment plan. The evidence supports the use of preemptive surgery for the management of highly selected metastatic CRC elderly patients.

The results of this study suggested that patients with a primary tumor located on the left side of the colon or rectum have a better prognosis than those with a primary tumor on the right side of the colon, which is consistent with the results of previous studies. A retrospective study by Corsini et al. aiming to study the effect of primary colorectal cancer tumor location on survival after pulmonary metastasectomy showed that left-sided colon and rectal cancer was associated with prolonged survival in patients after resection of PM (14). Yu et al. (15), using the Korean National Health Insurance database to study the prognostic factors of patients with colorectal cancer after PM resection, reported that the presence of distally located colon and rectal cancer is a positive factor for survival and prognosis. Yi, Chenghao (11) and Engstrand (16) also found that compared with the proximal colon, the distal colon and rectum were associated with better long-term survival after resection of metastatic tumors. All these results show that the primary tumor site has a good predictive effect on the outcome of patients after surgical resection. This discrepancy may be related to differences in the anatomical characteristics of the colorectal segments (17). More importantly, differences in molecular and pathological features reported in patients with right-sided and left-sided colon cancer may lead to different clinical features; for example, patients with metastatic right-sided cancer are more likely to have signet ring cell features, higher pathological T stage and grade, KRAS mutation, and microsatellite instability, which may also contribute to a worse prognosis of right-sided colon cancer (18–20). Currently, the TNM staging system is recommended for predicting the prognosis of CRC patients. In our study, patients with T1-2 stage disease had worse OS and CSS rates than those with T3 stage disease, which may be related to the pathological characteristics of the tumor itself or bias in the results due to the small sample of cases with stage T1-2 disease. Further clinical studies should be designed to study the association between T stage and prognosis in colorectal cancer patients with LM and/or PM.

Lymph node examination plays an important role in evaluating the quality of surgery and for pathological examination, which is associated with accurate staging and adjuvant treatment performance (21). Currently, the National Comprehensive Cancer Network (NCCN) and the American Society of Clinical Oncology (ASCO) recommend that at least 12 lymph nodes be examined. The current study also demonstrated that the number of regional lymph nodes examined is closely related to patient prognosis. When the number of regional lymph nodes examined is 12-20 or >20, the postoperative outcome of patients with LM and/or PM is significantly improved. Thus, surgeons should remove as many regional lymph nodes as possible to improve the prognosis of patients when resecting the primary tumor. Of course, we must also consider that more lymph node removal means greater surgical trauma. Importantly, no significant difference was observed in the prognosis of patients with different N stages, which is a novel and important finding of this study. Possible reasons include an insufficient number of lymph nodes examined to obtain an accurate N stage, and differences in disease status of distant metastatic organs, such as the size, number, and extent of metastases. More clinical research is needed to further investigate this finding.

The study by Yi et al. (11) found that among patients with single organ metastases of metastatic colorectal cancer, those with solitary pulmonary metastasis had the highest median OS. However, Siebenhüner et al. (22) reported that compared with patients undergoing resection of PM or liver and lung metastases, those with LM have better OS and CSS rates after metastatic tumors resected, which is consistent with our research results. These results show that the organ affected by distant metastasis also influences the long-term outcomes of patients after surgical resection.

In our study, race and primary tumor size were not significantly correlated with the prognosis of patients. A recent study by Feng et al. (23) using the SEER database to investigate the association between tumor size as a continuous variable and prognosis in nonmetastatic colon cancer suggested that there was a strong negative relationship between the primary tumor size and patient prognosis. However, this relationship was not found in this study. Yu et al. (15) reported that female sex was a positive prognostic factor for survival. However, our study found that male sex was a favorable factor for CSS. More clinical studies are still needed for further verification. Due to the limited patient information available in the SEER database, we were not able to study other factors that may affect prognosis. Some studies have reported that postoperative complication rates and mortality risk increased significantly when the primary tumor and synchronous liver metastases were resected simultaneously; therefore, staged operation should be recommended (24–26). However, a prospective study involving 84 patients found that when primary colorectal cancer and simultaneous liver metastases were resected at the same time, there appeared to be no difference in the complication rate. Delayed resection often compromises overall survival (27). Moreover, Zhang et al. (28) and Silberhumer et al. (29) also concluded that simultaneous surgical resection is a safe and effective treatment option for patients with CRC LM. Compared with staged surgery, there was no significant difference in the long-term prognosis of patients. At present, simultaneous surgical resection has become an optional treatment option for CRC patients with LM or PM. The lack of cytokeratin 20 expression in metastases is associated with poor overall survival for CRC PM patients (30). Isolated unilateral lung metastasis with normal CEA levels and no lymph node involvement is a positive prognostic factor for patients (31, 32). Another study reported that patients with hepatic regional lymph node involvement who underwent resection of CRC liver metastases had inferior survival compared to patients with negative nodes. Despite this poor prognostic factor, a small proportion of cases with involved nodes do achieve favorable long-term survival outcomes (33). For some CRC patients with LM and/or PM, surgical resection combined with chemotherapy or radiotherapy may also bring survival benefits (28, 34).

The study used the SEER database to explore the prognostic factors of patients with CRC LM and/or PM after surgical resection, and the results offer some very important insights and supporting evidence, providing a theoretical basis for clinical practice. Nevertheless, we must point out that this research still has some limitations. First, there was a lack of information about the patient’s general physical condition in the database, such as body mass index and comorbidities. Some studies have reported that patients with serious concomitant diseases often have a poor prognosis (10, 25). Second, the different levels of experience among surgeons can influence patient outcomes and may bias the results. Third, some important potential prognostic variables were not tested in this analysis. The SEER database contains information on the surgical treatments and general outcomes of the patients, but information on preoperative tumor markers, the extent of disease at the distant metastatic site, biological features such as microsatellite status, RAS-RAF mutations, adjuvant systemic and/or local-regional therapies is lacking, limiting further analyses of the possible factors affecting the prognosis of patients. Thus, the effect of selection bias could not be controlled. Finally, control of the indication for surgery, subjective definition of resectability, and access to tertiary care may influence the results of the study to some extent. Hence, we hope that a more complete public electronic database system can be established and that further clinical studies can be designed to overcome some of these problems to ensure that this evidence base is more comprehensive and reliable.

## Conclusion

For CRC patients with LM and/or PM who underwent resection of metastatic tumors, age < 65 years is associated with better long-term outcomes. Nevertheless, a significant number of elderly patients (≥65 years) may still benefit from surgical resection and achieve good long-term survival outcomes. Primary tumors located on the left side are positive prognostic factors for CRC patients with LM and/or PM compared with primary tumors located on the right side. When the primary tumor stage is T3, patients often can achieve better long-term survival, which should be further verified by more clinical studies. In addition, the number of regional lymph nodes examined appears to be positively correlated with long-term outcomes and compared with CRC patients with PM only or both PM and LM, patients with only LM have a better long-term prognosis.

## Data Availability Statement

Publicly available datasets were analyzed in this study. This data can be found here: Surveillance, Epidemiology, and End Results (SEER) database (https://seer.cancer.gov/).

## Author Contributions

PP and YC contributed to conception and design of the study. YL, PS, LW, XZ, YW, XC, PR, QL, YY, HC, HM, BS and XF contributed to the acquisition, analysis, or interpretation of data for the work. PP wrote the first draft of the manuscript. Then, YC critically revised this report. All authors contributed to the article and approved the submitted version.

## Funding

This study was supported by Sanming Project of Medicine in Shenzhen (No. SZSM201911012).

## Author Disclaimer

The contents of the present study are solely the responsibility of the authors. The funders had no role in study design, data collection and analysis, decision to publish or preparation of the manuscript.

## Conflict of Interest

The authors declare that the research was conducted in the absence of any commercial or financial relationships that could be construed as a potential conflict of interest.

## Publisher’s Note

All claims expressed in this article are solely those of the authors and do not necessarily represent those of their affiliated organizations, or those of the publisher, the editors and the reviewers. Any product that may be evaluated in this article, or claim that may be made by its manufacturer, is not guaranteed or endorsed by the publisher.
